# Influence of Leptin and Adiponectin Supplementation on Intraepithelial Lymphocyte and Microbiota Composition in Suckling Rats

**DOI:** 10.3389/fimmu.2019.02369

**Published:** 2019-10-09

**Authors:** Blanca Grases-Pintó, Mar Abril-Gil, Margarida Castell, Maria J. Rodríguez-Lagunas, Stephen Burleigh, Frida Fåk Hållenius, Olena Prykhodko, Francisco J. Pérez-Cano, Àngels Franch

**Affiliations:** ^1^Physiology Section, Department of Biochemistry and Physiology, Faculty of Pharmacy and Food Science, University of Barcelona, Barcelona, Spain; ^2^Research Institute of Nutrition and Food Safety of the University of Barcelona (INSA-UB), Santa Coloma de Gramenet, Spain; ^3^Food for Health Science Centre, Lund University, Lund, Sweden; ^4^Department of Food Technology, Engineering and Nutrition, Lund University, Lund, Sweden

**Keywords:** leptin, adiponectin, suckling rat, IEL, microbiota

## Abstract

Dietary components in early life play a role in both microbiota and intestinal immune system maturation in mammalian species. Adipokines, as endogenously produced hormones from breast milk, may have an impact on this process. The aim of the present study was to establish the influence of leptin and adiponectin supplementation during suckling on the intraepithelial lymphocyte composition, intestinal barrier function, intestinal gene expression, and gut microbiota in rat. For this purpose, newborn Wistar rats were supplemented daily with leptin, adiponectin, or whey protein concentrate during the first 21 days of life. Lymphocyte composition was established by immunofluorescence staining and flow cytometry analysis; intestinal gene expression by real-time PCR and cecal microbiota were analyzed through 16S rRNA gene sequencing. Although leptin and adiponectin were able to increase the Tc TCRαβ^+^ and NKT cell proportion, they decreased the NK cell percentage in IEL. Moreover, adipokine supplementation differentially modified CD8^+^ IEL. While the supplementation of leptin increased the proportion of CD8αα^+^ IEL (associated to a more intestinal phenotype), adiponectin enhanced that of CD8αβ^+^ (related to a peripheral phenotype). Furthermore, both adipokines enhanced the gene expression of TNF-α, MUC-2, and MUC-3, and decreased that of FcRn. In addition, the adipokine supplementations decreased the abundance of the Proteobacteria phylum and the presence of *Blautia*. Moreover, leptin-supplemented animals had lower relative abundance of *Sutterella* and a higher proportion of *Clostridium* genus, among others. However, supplementation with adiponectin resulted in lower abundance of the *Roseburia* genus and a higher proportion of the *Enterococcus* genus. In conclusion, the supplementation with leptin and adiponectin throughout the suckling period had an impact on both the IEL composition and the gut microbiota pattern, suggesting a modulatory role of these adipokines on the development of intestinal functionality.

## Introduction

The intestinal microbiota develops in early life and although the microbiota colonizes the intestine mainly after birth, over the years the opinion about when the colonization starts has been changing (1). In the past, it was considered that the *in utero* environment was sterile and it was not until delivery that colonization began, when the newborn passed through the vagina or skin (Cesarean section) and adopted maternal microbiota (2). However, latest studies have shown that bacteria may already start their colonization during pregnancy, since microbiota have been found to present in the placenta or amniotic fluid, and have even been isolated in the meconium (2–4). It is also suggested that the placenta has its own microbiota (5). Despite all the factors affecting the gut microbiome *in utero*, the main factor shaping the gut ecosystem after birth is diet, when bacteria are transferred to the infant through breast milk and later with the introduction of solid food (1). The process of gut colonization in newborns sets the stage for adults' microbiome and its dysregulation in early life could affect health thereafter (6).

Intestinal microbiota composition during the first year of life differs from that present in adults, it is less stable, more variable in its composition and has low diversity (7, 8). At birth, the newborn's gut has an aerobic environment, where mainly facultative anaerobes such as *Enterobacteriaceae* or *Staphylococcus* can colonize (9, 10). Some days after, the growth of *Bifidobacterium, Clostridium*, and *Bacteroidetes*, strict anaerobe bacteria, appear due to the conversion of the intestine to an anaerobic environment (11). Until weaning, the microbiota depends on the diet of the infant, thus breastfed infants have *Bifidobacteria* as dominant genus, whereas formula-fed newborns show an increase in the *Enterobacteria* (2). When solid food is introduced, the microbiota suffers another important change and it turns toward adult-like microbiota, presenting an increase in the abundance of *Bacteroides, Clostridium, Ruminococcus*, and *Prevotella* (9, 10). Moreover, there are other different factors that influence the intestinal microbiota of the newborn, such as the gestational age, the birth weight, gender of the baby, mode of delivery, location of birth (home or hospital), geographic location, hospitalization of the newborn, maternal and/or infant use of antibiotics, probiotics or prebiotics; finally, the size of the family or the presence of pets at home could modulate it (12).

Microbiota plays an important role in the maturation of the immune system, mainly with those lymphocytes present in the gut-associated lymphoid tissue (GALT) (9). At birth, GALT is not fully mature and it is still developing during suckling (13). In the epithelium of the intestinal mucosa are located the intraepithelial lymphocytes (IEL), which are the first immune cells in contact with intestinal antigens, protecting the gut from infections (14). The importance of the crosstalk between the colonizing bacteria and the underlying lymphoid cells is well-known (15). If there is not a good development of the GALT or a good intestinal colonization by the microbiota, this communication is lost and makes the baby more susceptible to infections (15, 16).

Human milk provides a high number of bioactive factors that promote the formation of innate and adaptive immune factors, such as lactoferrin, cytokines, growth factors, adipokines, secretory immunoglobulin A (sIgA), among others. These components protect the infant against infections and promote their immune system maturation (17). Moreover, breast milk also contains bacteria from the mother's gut and some compounds, such as prebiotic oligosaccharides, which also participate in the microbiota's development (18). In previous studies, we have described the role of leptin and adiponectin, adipokines present in breast milk, in the maturation of the early life immune system, particularly in systemic immune response (19) and also in the inductive GALT (20). In line with this, we hypothesized that these adipokines could also influence the effector GALT, the gut microbiota and also their interaction, promoting the development in this first stage of life. Thus, the purpose of this paper is to study the influence of leptin and adiponectin supplementation on the IEL composition, intestinal gene expression and microbiota pattern in suckling rats.

## Materials and Methods

### Animals

Pregnant Wistar rats (G15) were obtained from Janvier Labs (Le Genest Saint Isle, France). Dams were housed in individual cages and fed with commercial diet corresponding to the American Institute of Nutrition 93M formulation (Harlan Teklad, Madison, Wisconsin, USA) and water *ad libitum*. The animals were maintained under controlled temperature and humidity conditions in a 12:12 h light:dark cycle in the animal facilities of the Faculty of Pharmacy and Food Science. The gestating rats were monitored daily and allowed to deliver at term. Proportion of males and females were between 40 and 60% in each litter. The day after birth was registered as day 1 of life. Litters were unified to 9 pups per lactating dam and had free access to the breast milk and rat diet. Daily, pups were separated from their mother, to be identified, weighted, and orally administered. To avoid the influence of biological rhythms, handling was done in the same time range.

With regard to sample size estimation, because of the variability among litters, four litters were required for each group. This calculation was made by the Appraising Project Office's program from the Universidad Miguel Hernández de Elche (Alicante), used to provide statistically significant differences among groups, assuming that there is no dropout rate and considering a type I error of 0.05 (two-sided). The studies were performed in accordance with the criteria outlined by the Guide for the Care and Use of Laboratory Animals. Experimental procedures were reviewed and approved by the Ethical Committee for Animal Experimentation of the University of Barcelona and the Ethical Committee of the Generalitat de Catalunya (CEEA/UB 220/15 and DAAM: 8521).

### Dietary Supplementation

Suckling rats were randomly distributed into four groups, according to the oral supplementation received: the Reference, the Leptin, the Adiponectin, and the Whey Protein Concentrate (WPC) groups. Each group was constituted by four different litters (*n* = 36 pups/group). Animals from the Reference group were administered with the same volume of vehicle (mineral water) as the supplemented groups (10 mL/kg/day). The Leptin group was supplemented with a solution of leptin (PeproTech®, Rocky Hill, NJ, USA) at a dose of 0.7 μg/kg/day in mineral water, the Adiponectin group was supplemented with adiponectin (PeproTech®) solution at a dose of 35 μg/kg/day in mineral water and the WPC group was supplemented with Lacprodan® MFGM-10 (Arla Foods Ingredients Group, Diby, Denmark) at a dose of 0.3 g/kg/day in mineral water. All these doses have been demonstrated in previous studies to have immunomodulatory activity (19, 20). The WPC group was used as positive control of modulation by breast milk bioactive factors. Suckling rats were daily supplemented during the whole lactating period (21 days), using low-capacity syringes (Hamilton Bonaduz, Bonaduz, Switzerland) adapted to oral 25- or 23-gauge gavage tubes and 27 mm in length (ASICO, Westmont, IL, USA), as previously described (21). To allow gastric emptying, litters were separated from their dams half an hour before oral supplementation.

### Experimental Design 1

On day 10 -in the middle of the suckling period-, the functionality of the intestinal epithelial barrier was assessed on 3 rats of each litter (*n* = 12/group) by an *in vivo* permeability assay, as previously described (22). Briefly, a solution of 4 kDa-dextran conjugated to FITC (Sigma-Aldrich) was orally administered to rats using low-capacity syringes adapted to oral gavage tubes. There was an additional group of animals that was only administered with an equivalent volume of PBS (10 mL/kg) to rule out the fluorescent background of the plasma samples. After 4 h of the dextran administration, the animals were euthanized and plasma was obtained, diluted and the fluorescence emission was quantified by triplicate at an excitation wavelength of 490 nm in the Modulus™ microplate spectrophotometer (Turner Biosystems, Sunnyvale, CA, USA). Moreover, a 0.5 cm portion of the middle intestine was immediately conserved in RNAlater® (Applied Biosystems, Weiterstadt, Germany), incubated at 4°C overnight and stored at −20°C until PCR analysis.

### Experimental Design 2

At the end of the suckling period (day 21), another 3 rats of each litter (*n* = 12/group) were anesthetized intramuscularly with ketamine (90 mg/kg) (Merial Laboratories S.A., Barcelona, Spain) and xylazine (10 mg/kg) (Bayer A.G., Leverkusen, Germany), exsanguinated and the small intestine was carefully collected. After discarding the duodenum, a 0.5 cm portion of the middle intestine was immediately conserved, as previously on day 10, until PCR analysis. The proximal 1/3 portion of the intestine was opened lengthwise along the mesenteric line and was cut into 2 cm pieces and immersed in Hanks balanced salt solution (HBSS) (Sigma-Aldrich, Madrid, Spain) supplemented with 100 IU/mL streptomycin–penicillin (Sigma-Aldrich) and 5% fetal bovine serum (FBS; Sigma-Aldrich) until further steps in IEL isolation. Moreover, cecal content was also collected and kept at −80°C for gut microbiota analysis.

### Intraepithelial Lymphocyte Isolation

IEL suspensions were obtained following procedures previously established in our laboratory (23), with some modifications. Briefly, small pieces of intestine were incubated with a 1 mM 1,4-dithiothreitol (DTT) (Sigma-Aldrich) solution in HBSS with 10% FBS under continuous agitation (55 u/min, 20 min, 37°C). The first supernatants were collected by decanting the tubes. Afterwards, a 5 mM ethylenediaminetetraacetic acid (EDTA) (Sigma-Aldrich) solution in HBSS with 10% FBS was added to the remaining intestinal tissue and incubated twice under continuous agitation (55 u/min, 15 min, 37°C). The supernatants from each step were decanted from the tissue pieces, collected and then centrifuged (538 g, 5 min, 4°C). The resulting pellet containing IEL and epithelial cells from all steps was then subjected to IEL purification.

### Intraepithelial Lymphocyte Purification

The pellet containing IEL was resuspended in 44% Percoll® (Sigma-Aldrich). Each cell suspension was overlaid on 67.5% Percoll® and, after centrifugation (600 g, 30 min, room temperature), viable lymphocytes were recovered from the interface. Finally, purified lymphocytes were resuspended in Roswell Park Memorial Institute (RPMI) 1640 medium (Sigma-Aldrich) enriched with 10% FBS, 100 IU/mL streptomycin–penicillin, 2 mM L-glutamine (Sigma-Aldrich) and 0.05 mM 2-β-mercaptoethanol (Merck Millipore). Cell number and viability were determined using Countess^TM^ Automated Cell Counter (Invitrogen^TM^, Thermo Fisher Scientific, Barcelona, Spain).

### Immunofluorescence Staining and Flow Cytometry Analysis

IEL (2 × 10^5^) were stained with anti-rat monoclonal antibodies (mAb) conjugated to fluorescein isothiocyanate (FITC), phycoerythrin (PE), peridininchlorophylla protein (PercP), allophycocyanin (APC) or APC-cyanine (Cy)7, as in previous studies (24). In this case, the mAb used were anti-CD4, anti-CD8α, anti-CD8β, anti-TCRαβ, anti-TCRγδ, anti-NKR-P1A, anti-CD25, anti-CD45RA (BD Biosciences, San Diego, USA), anti-CD62L, and anti-CD103 (Biolegend, San Diego, CA, USA). After standard procedures (20), samples were analyzed using a Gallios^TM^ Cytometer (Beckman Coulter Inc., Miami, FL, USA) in the Scientific and Technological Centers of the University of Barcelona (CCiT-UB). The results were assessed by FlowJo version 10 software (TreeStar, Inc., Ashland, OR, USA). Lymphocyte subsets were defined as follows: Th (TCRαβ^+^ NKR-P1A^−^ CD4^+^) cells; Tc TCRαβ^+^ (TCRαβ^+^ NKR-P1A^−^ CD8^+^) cells; Tc TCRγδ^+^ (TCRγδ^+^ CD8^+^) cells; natural killer (NK) (NKR-P1A^+^ TCRαβ^−^) cells; NKT (NKR-P1A^+^ TCRαβ^+^) cells; CD4^+^ (CD4^+^ CD8^−^) cells; CD8^+^ (CD4^−^ CD8^+^) cells; CD8αα^+^ (CD8α^+^ CD8β^−^); CD8αβ^+^ (CD8α^+^ CD8β^+^). Results are expressed as percentages of positive cells in the lymphocyte population selected according to their forward-scatter cells (FSC) and side-scatter characteristics (SSC) or in a particular selected population.

### Quantification of Gene Expression in Small Intestine

For RNA isolation, small intestine samples from 6 rats/group randomly selected conserved in RNAlater® were processed as in previous studies (25). Tissue samples were homogenized in a FastPrep (MP Biomedicals, Illkirch, France) for 30 s. RNA was obtained using a RNeasy Mini Kit (Qiagen, Madrid, Spain) following the manufacturer's instructions. RNA purity and concentration were determined by NanoPhotometer (BioNova Scientific, S.L. Fremont, CA, USA). Thereafter, cDNA was obtained using TaqMan® Reverse Transcription Reagents (Applied Biosystems).

The specific PCR TaqMan® primers (Applied Biosystems) used to perform the PCR quantitative assay were: *Tlr2* [Rn02133647_s1, inventoried (I)], *Tlr4* (Rn00569848_m1, I), *Tlr5* (Rn04219239_s1, I), *Tlr7* (Rn01771083_s1, I), *Tlr9* (Rn01640054_m1, I), *Infg* (Rn00594078_m1, I), *Tnf* (Rn99999017_m1, I), *Il10* (Rn00563409_m1, I), *Muc2* (Rn01498206_m1, I), *Muc3* (Rn01481134_m1, I), *Prdm1* (Rn03416161_m1, I, encoding for Blimp-1), *Fcgrt* (Rn00583712_m1, I, encoding for FcRn), *Pigr* (Rn00562362_m1, I), *Cldn2* (Rn02063575_s1, I), *Cldn4* (Rn01196224_s1, I), *Ocln* (Rn00580064_m1, I), and *Tjp1* (Rn02116071_s1, I, ZO-1). Quantification of the studied genes was normalized to the *Gusb* (β-glucuronidase, Rn00566655_m1, I) housekeeping gene, which showed constant level of expression in our experimental conditions and a similar level of expression to the studied genes. Real-time PCR assays were performed in duplicate using an ABI Prism 7900HT sequence detection system (Applied Biosystems). The SDS software (version 2.4, Applied Biosystems) was used to analyze the expression of data.

The amount of target mRNA relative to the endogenous control expression was calculated for the three nutritional intervention groups relative to values from the Reference group, which represents 100% of the gene expression using the 2^−ΔΔ*Ct*^ method, as previously described (26). Results are expressed as percentage of values of each supplemented group normalized to the mean value obtained for the Reference group, which was set at 100%.

### DNA Extraction

Cecal DNA was extracted using the QIAamp DNA Stool Mini Kit (Qiagen), as previously described (27). The protocol from the manufacturer was followed with an addition of a bead beating step. Sterile glass beads (sized 1 mm Ø) were added together with stool lysis buffer to the samples and cell lysis was performed for 4 min at 25 Hz using a TissueLyser (Qiagen), followed by a heating step at 95°C for 5 min. After lysis, DNA-damaging substances and PCR inhibitors were neutralized using an InhibitEX tablets (provided with the kit) and the DNA was purified on QIAamp Mini spin columns.

### PCR Amplification of the V4 Region of Bacterial 16S rRNA Genes

The V4 region of 16S rRNA gene was amplified by PCR with forward and reverse primers according to Kozich et al. (28), containing Illumina (Illumina Inc., San Diego, CA, USA) adapter sequences and unique dual indexes used to tag each PCR product, according to the 16S-protocol provided by Illumina. Primer sequences can be found in Table 1. Briefly, PCR was carried out in 25 μL reactions with 0.2 μM forward and reverse primers, with 12.5 ng template DNA and 12.5 μL of 2 × KAPA HiFi HotStart Ready Mix kit (KAPA Biosystems, Woburn, MA, USA). Thermal cycling consisted of initial denaturation at 95°C for 3 min followed by 25 cycles of denaturation at 95°C for 30 s, annealing at 55°C for 30 s, and extension at 72°C for 30 s, followed by a final step of 72°C for 5 min. The amplicon products were purified with Agencourt AMPureXP Kit (Beckman Coulter, Miami, FL, USA). Next, a second PCR was thereafter performed to attach Illumina adapters and unique dual indexes to each sample, followed by a clean-up step with AmPureXP Kit (Beckman Coulter). PCR amplicons were visualized using 0.1% agarose gel electrophoresis to verify the size of the amplicon. Negative extraction controls did not produce visible bands.

**Table 1:** Primer sequences for amplification of V4 region of 16S rRNA gene.

515f Forward primer with Illumina overhang adaptor (underlined)	5^′^TCGTCGGCAGCGTCAGATGTGTATAAGAGACAGGTGCCAGCMGCCGCGGTAA
806r Reverse primer with Illumina overhang adaptor (underlined)	5^′^GTCTCGTGGGCTCGGAGATGTGTATAAGAGACAGGGACTACHVGGGTWTCTAAT

*The underlined part represents the adapters for Illunima MiSeq instrument*.

### Amplicons Quantitation, Pooling, and Sequencing

Amplicon DNA concentrations were quantified using the Qubit4.0 Fluorometer (Life Technologies, Stockholm, Sweden). Amplicons were combined in equimolar ratios into a single tube with a final concentration of the DNA library of 4 pM. As an internal control, 5% of PhiX was added to the amplicon pool. Paired-end sequencing with a read length of 2 × 250 bp was carried out on a Miseq Instrument (Illumina) using a Miseq reagent kit v2 (Illumina).

### Sequence Analysis

Sequenced data were analyzed with an open-source bioinformatics pipeline Quantitative Insights into Microbial Ecology (QIIME), which allows the analysis of high-throughput community sequencing data. Sequences were removed when lengths were <200 nucleotides, contained ambiguous bases, primer mismatches or if the homo polymer runs were in excess of six bases. After quality filtering, a total of 3,913,202 reads were obtained for the 29 samples with an average of 134,938 reads per sample (min: 64,101 and max: 232,472). Similar sequences were binned into operational taxonomic units (OTU) using UCLUST, with a minimum of 97% sequence similarity. The most abundant sequence in each OTU was chosen to represent the respective OTU. Then, representative sequences (most abundant) from each OTU were aligned using Python Nearest Alignment Space Termination (PyNAST). Taxonomy was assigned using Greengenes database (v.13_8). The heat map of the mean relative abundances of genera was also obtained from QIIME. In addition, the relative proportion of the bacteria was calculated using GraphPad Prism software (version 7.0, GraphPad Software, La Jolla, CA, USA). Finally, Venn diagrams were made by Bioinformatics and Evolutionary Genomics program from the University of Ghent.

### Statistical Analysis

The software IBM Statistical Package for the Social Sciences (SPSS, version 22.0, Chicago, IL, USA) was used to perform statistical analysis. Levene's and Shapiro–Wilk tests were applied to assess variance equality and normal distribution, respectively. Conventional one-way ANOVA test was performed considering the experimental group as the independent variable. When supplementation had a significant effect on the dependent variable, Bonferroni *post-hoc* test was applied. Otherwise, non-parametric Kruskal–Wallis test followed by the *post-hoc* Mann–Whitney *U*-test were used in order to assess significance for independent and related samples, respectively. A parallel analysis was performed taking into account the sex of the animals as variable. As no sexual dimorphism in the variables analyzed here in the early life animal was found, animals in each group were considered together independently of sex. Significant differences were accepted when *p* < 0.05.

## Results

### Intestinal Barrier Function

To study the intestinal epithelial barrier function on day 10, when it is still in early development, an *in vivo* permeability assay was performed to determine the paracellular pass of the 4 kDa-dextran labeled with FITC. No effect due to any of the supplementations was found, suggesting that the compound tested do not act through the modulation of such function (Supplementary Figure 1A).

### Intraepithelial Lymphocyte Composition

**Figure 1:**
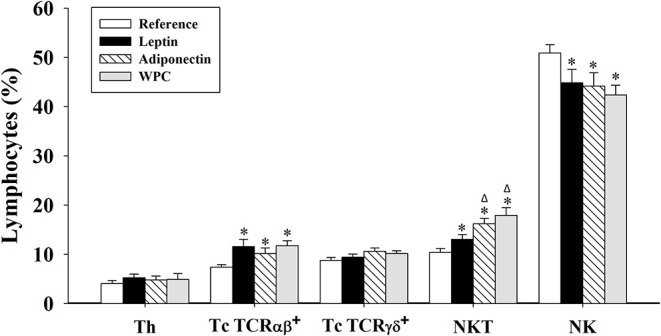
Main IEL subsets at day 21 from the four groups: Reference, Leptin, Adiponectin, and Whey Protein Concentrate (WPC). Lymphocyte populations were defined as follows: Th (TCRαβ^+^ NKR-P1A^−^ CD4^+^) cells; Tc TCRαβ^+^ (TCRαβ^+^ NKR-P1A^−^ CD8^+^) cells; Tc TCRγδ^+^ (TCRγδ^+^ CD8^+^) cells; NKT (NKR-P1A^+^ TCRαβ^+^) cells; and natural killer (NK) (NKR-P1A^+^ TCRαβ^−^) cells. Results are expressed as mean ± S.E.M. (*n* = 8–12 pups). Statistical differences: *p < 0.05 vs. Reference group, ^Δ^*p* < 0.05 vs. Leptin group.

The percentage of the main subsets present in IEL was determined at day 21 (Figure 1). After the supplementation, during the whole suckling period, both leptin and adiponectin were able to increase the Tc TCRαβ^+^ and NKT proportions and to decrease NK cell percentage in IEL compared to the Reference group (*p* < 0.05, Figure 1). The WPC group showed the same effect (*p* < 0.01), indicating that the effect of the adipokines on the IEL population is in line with that of the whey protein, containing a high concentration of bioactive factors with immunomodulatory effects. No changes due to diets were observed in Th or Tc TCRγδ^+^.

**Figure 2:**
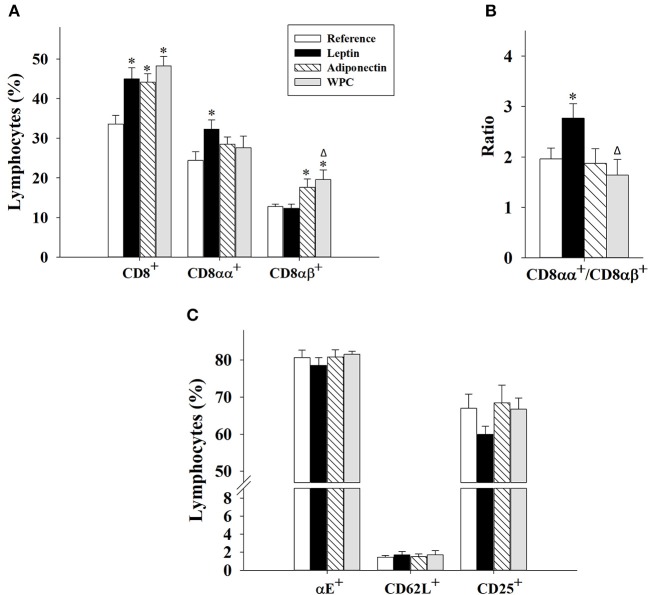
CD8^+^ IEL subsets **(A,B)** and surface expression of some molecules in IEL **(C)** at day 21 from the four groups: Reference, Leptin, Adiponectin, and Whey Protein Concentrate (WPC). Percentage of CD8^+^ (CD8^+^ CD4^−^); CD8αα^+^ (CD8α^+^ CD8β^−^); and CD8αβ^+^ (CD8α^+^ CD8β^+^) **(A)**. The ratio CD8αα^+^/CD8αβ^+^ **(B)**. Percentage of αE^+^ integrin (CD103^+^), CD62L^+^ selectin, and CD25^+^ IEL **(C)**. Results are expressed as mean ± S.E.M. (*n* = 8–12 pups). Statistical differences: *p < 0.05 vs. Reference group, ^Δ^*p* < 0.05 vs. Leptin group.

With regard to the CD8 co-receptor, all components enhanced its presence after 21 days of supplementation (*p* < 0.01 vs. Reference group, Figure 2A). However, leptin and adiponectin differentially modulated CD8^+^ IEL development. Changes induced by leptin supplementation were associated with an increase in a more intestinal phenotype (CD8αα^+^) (*p* < 0.05 vs. Reference group), whereas adiponectin or WPC administration was related with a more peripheral phenotype (CD8αβ^+^, *p* < 0.05 vs. Reference and Leptin group, Figure 2A). These alterations caused higher CD8αα^+^/CD8αβ^+^ ratio in animals supplemented with leptin compared to reference (*p* < 0.05, Figure 2B).

Furthermore, the expression of intestinal homing molecules (αE integrin and CD62L selectin) and the activation marker CD25 was also studied (Figure 2C). None of the dietary interventions during 21 days was able to modify the expression of any of the three molecules.

### Intestinal Gene Expression

**Figure 3:**
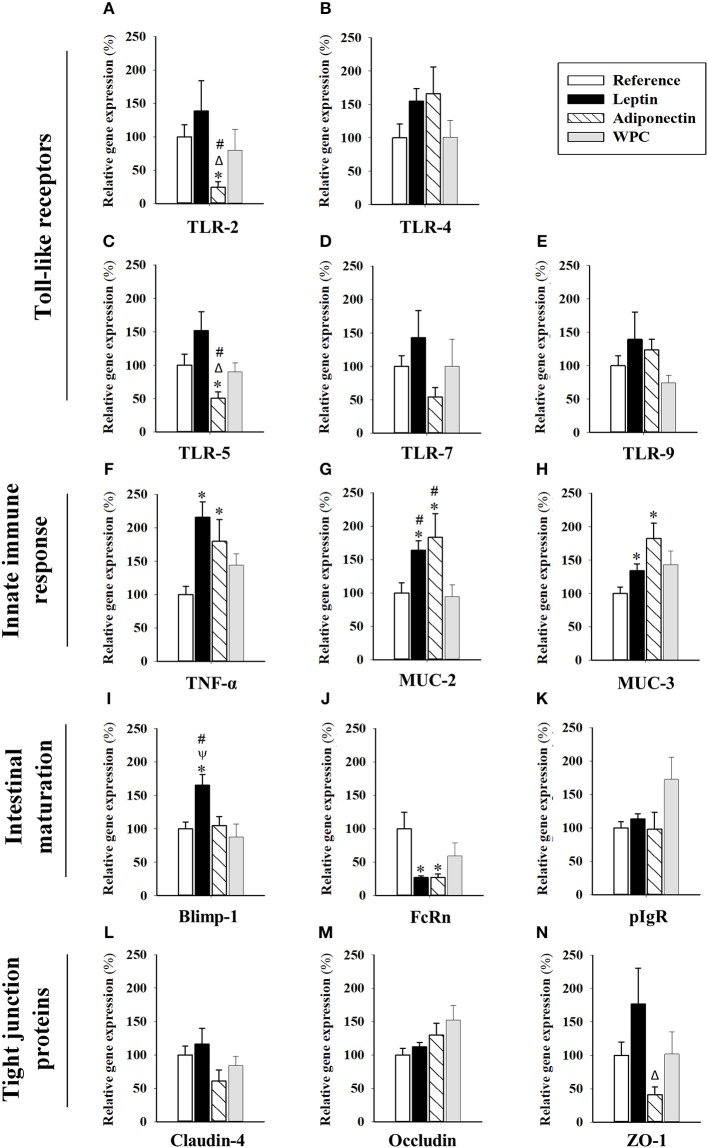
Gene expression in small intestinal samples from the four groups: Reference, Leptin, Adiponectin, and Whey Protein Concentrate (WPC). TLR-2 **(A)**, TLR-4 **(B)**, TLR-5 **(C)**, TLR-7 **(D)**, TLR-9 **(E)**, TNF-α **(F)**, MUC-2 **(G)**, MUC-3 **(H)**, Blimp-1 **(I)**, FcRn **(J)**, pIgR **(K)**, Claudin-4 **(L)**, Occludin **(M)**, and ZO-1 **(N)**. Results are expressed as mean ± S.E.M. (*n* = 6–12 pups). Statistical differences: *p < 0.05 vs. Reference group, ^Δ^*p* < 0.05 vs. Leptin group, ^**ψ**^*p* < 0.05 vs. Adiponectin group, ^#^*p* < 0.05 vs. WPC group.

The relative gene expression in the intestine of molecules associated with the bacteria host cell crosstalk [Toll-like receptor (TLR)-2, TLR-4, TLR-5, TLR-7, TLR-9], innate immune response [tumor necrosis factor (TNF)-α, mucin (MUC)-2, MUC-3], intestinal maturation and function [B-lymphocyte-induced maturation-protein-1 (Blimp-1), neonatal-Fc-receptor (FcRn), polymeric Ig receptor (pIgR)], and molecules related to tight junctions of the epithelial barrier [Claudin-4, Occludin, zona occludens (ZO)-1], are shown in Figure 3. Moreover, other molecules such as interferon (IFN)-γ, interleukin (IL)-10, and Claudin-2 were also studied without reaching levels of detection.

With regard to the TLR expression, although no changes were observed in TLR-4 and TLR-9 (Figures 3B,E, respectively), adiponectin supplementation, but not leptin, showed a tendency to decrease TLR-7 (Figure 3D) and significantly decreased TLR-2 and TLR-5 gene expression (*p* < 0.05 vs. Reference, Leptin and WPC groups, Figures 3A,C).

Focusing on molecules related to the intestinal innate immune response, TNF-α, MUC-2, and MUC-3, their gene expression was higher in those suckling rats supplemented with both adipokines (*p* < 0.05, Figures 3F–H), suggesting a role in the early development of innate immunity. These changes were already observed only for MUC-2 on day 10 (*P* < 0.05, Supplementary Figure 1B). In addition, leptin dietary intervention promoted intestinal maturation by means of enhancing the Blimp-1 gene expression (*p* < 0.01, Figure 3I). Moreover, both adipokine supplementations during 21 days lowered the expression of FcRn gene (*p* < 0.05, Figure 3J). On the other hand, none of the three compounds were able to modify the gene expression of pIgR (Figure 3K) Neither Blimp-1 nor FcRn gene expression was modified earlier in life due to the adipokines supplementation (day 10, Supplementary Figure 1B).

Regarding the molecules related to the tight junctions of the epithelial barrier, although no differences were detected on day 10 (Supplementary Figure 1B), the animals supplemented with adiponectin showed lower gene expression of ZO-1 (*p* < 0.05 vs. Leptin group, Figure 3N). However, no differences in Claudin-4 or Occludin due to adipokine supplementation were observed on both studied days (Figures 3L,N and Supplementary Figure 1B).

### Gut Microbiota Composition

**Figure 4:**
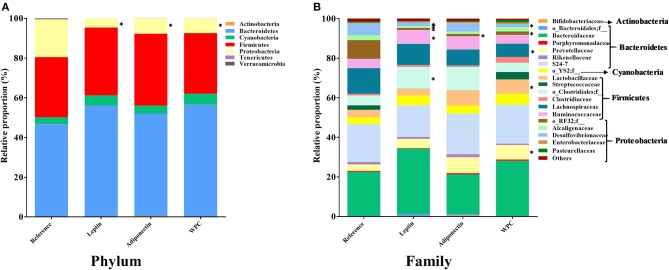
The relative proportion of the bacteria was calculated for phylum **(A)** and family **(B)** at day 21 from the four groups: Reference, Leptin, Adiponectin, and Whey Protein Concentrate (WPC) (*n* = 6–12 pups). Statistical differences: *p < 0.05 vs. Reference group.

At the end of the study, cecal microbiota composition was established. To assess the diversity of microbial populations, Shannon-Wiener and CHAO1 indexes were calculated. None of the four groups showed any significant change in the microbiota diversity (Table 2). However, the dietary interventions influenced the microbiota composition at different taxonomic levels. The main phylum of bacteria present in the Reference group was Bacteroidetes, followed by Firmicutes, Proteobacteria and Cyanobacteria, and were approximately 47, 30, 19, and 3% of total gut microbiota, respectively (Figure 4A). The three supplementations induced lower Proteobacteria phylum proportion compared to the Reference group (*p* < 0.01, Figure 4A). In the Leptin group, this decrease was due to the lower abundance of *Desulfovibrionaceae, Alcaligenaceae*, and the unclassified family from the *RF32* order (*p* < 0.01 vs. Reference group, Figure 4B). In the case of the Adiponectin group, this change was caused only by a lower presence of the unclassified family from the *RF32* order (*p* < 0.01 vs. Reference group, Figure 4B) and WPC also showed less *Desulfovibrionaceae* and unclassified family from *RF32* order than the Reference group (*p* < 0.01, Figure 4B). The Firmicutes/Bacteroidetes ratio was also assessed without changes observed among groups (Table 2). Although no significant changes were observed in Bacteroidetes and Firmicutes phylum proportions due to the interventions, some modifications in their respective families were observed in animals supplemented with leptin and WPC. In this sense, the Leptin group had a higher proportion of *Ruminococcaceae* and the unclassified family from the *Clostridiales* order (*p* < 0.01 vs. Reference group, Figure 4B). On the other hand, an increase in the *Prevotellaceae* and *Lactobacillaceae* families and a decrease in the *Lachnospiraceae* family proportions were observed in those animals fed with WPC compared to the Reference group (*p* < 0.05, Figure 4B).

**Table 2:** Shannon-Wiener and CHAO1 indexes from the four groups: Reference, Leptin, Adiponectin, and Whey Protein Concentrate (WPC) at the end of the suckling period.

	**Reference**	**Leptin**	**Adiponectin**	**WPC**
Chao 1	190.00 ± 26.40	222.74 ± 42.86	213.00 ± 37.22	182.50 ± 20.66
Shannon	5.02 ± 0.17	5.04 ± 0.25	5.16 ± 0.34	4.76 ± 0.27
Firmicutes/Bacteroidetes ratio	0.58 ± 0.19	0.57 ± 0.19	0.67 ± 0.17	0.68 ± 0.09

*The results are expressed as a mean ± S.E.M. (n = 9–12)*.

**Figure 5:**
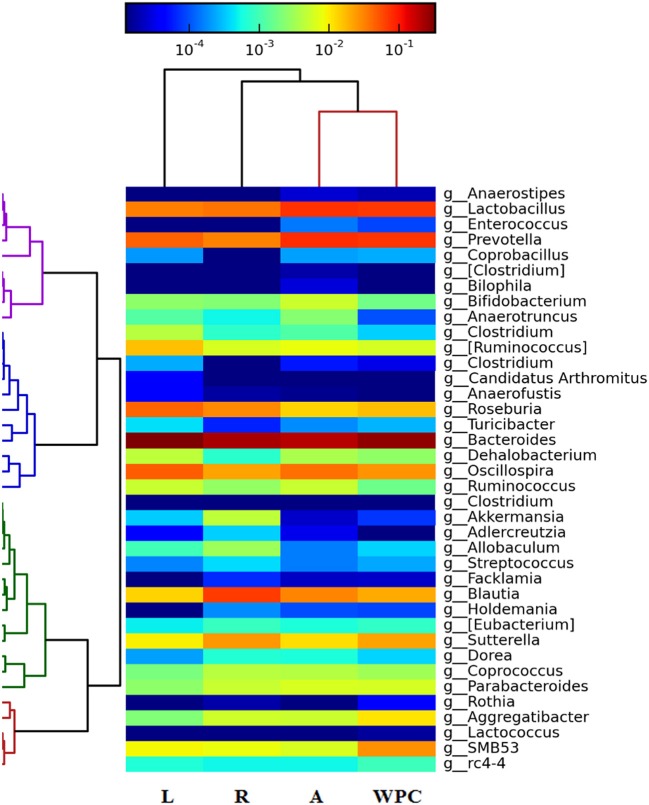
Heatmap showing the abundance of microbiota at genus level at day 21 from the four groups: Reference (R), Leptin (L), Adiponectin (A), and Whey Protein Concentrate (WPC) (*n* = 6–12 pups). Intense blue and red shades indicate lower and higher relative abundances, respectively.

The heatmap of Figure 5 shows the changes found at genus level. The regions with a higher presence of a particular genus are indicated in red and those with a lower presence in blue. All supplementations were able to decrease the relative abundance of *Blautia* genus of the cecal microbiota compared to the Reference group (*p* < 0.01 for the Leptin and WPC groups and *p* < 0.05 for the Adiponectin group, Figure 5). Moreover, some specific effects of each adipokine were observed. On the one hand, rats fed with leptin showed a higher proportion of the *Clostridium* and *Dehalobacterium* genera and a lower presence of the *Holdemania* and *Sutterella* genera (*p* < 0.01 vs. Reference group). On the other hand, an increase of the *Enterococcus* genus and a decrease in the *Roseburia* and *Allobaculum* genera abundances were observed in the Adiponectin group (*p* < 0.05). WPC nutritional intervention promoted the presence of the *Dehalobacterium* genus and reduced the relative abundance of the *Anaerotruncus* and *Adlercreutzia* genera (*p* < 0.05, Figure 5).

**Figure 6:**
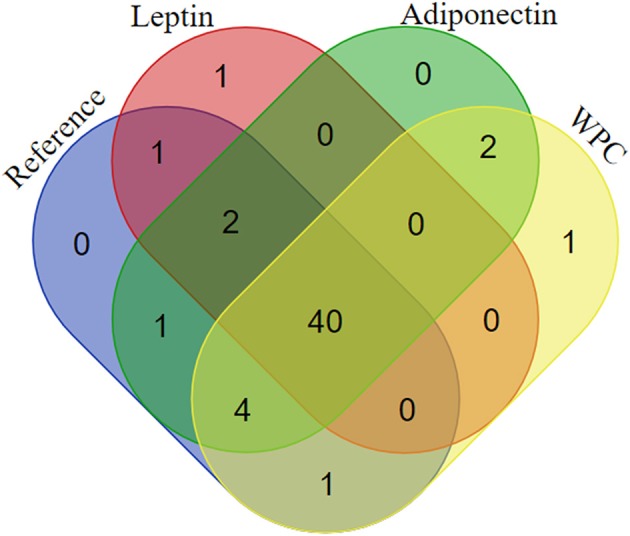
Venn diagram showing the distribution of the genera in the four groups: Reference, Leptin, Adiponectin, and Whey Protein Concentrate (WPC) (*n* = 6–12 pups).

In order to better understand the presence or the absence of bacterial genera found in each group, Venn diagrams were plotted (Figure 6). Among all cecal genera detected, 40 genera were present in the four groups studied. Nevertheless, the supplementation with leptin promoted the specific colonization with the *Candidatus arthromitus* species whereas the *Lactococcus* genus appeared only after the supplementation with WPC (Figure 6).

## Discussion

In previous studies, we demonstrated that a daily supplementation with leptin or adiponectin during the whole suckling period was able to enhance the immune system of pups, not only in the intestinal compartment (20), but also at systemic level (19). These results prompted us to further study the influence of the supplementation of these adipokines on the IEL subsets, as representative effector cells of GALT, intestinal gene expression and cecal microbiota composition. Moreover, as WPC has previously shown immunomodulatory effects on neonatal rats, one of the groups received it as a positive control of modulation by breast milk bioactive factors. However, a limitation of the study is the adiponectin structure used. Whereas, the most abundant form of adiponectin in human milk is the high molecular weight multimer (29), we have supplemented the rats with the monomeric form. However, and although leptin and adiponectin plasmatic levels in pups were not assessed after supplementation in this approach, their intestinal absorption in rodents has previously been described (30, 31).

First of all, on day 10 of the suckling period, the intestinal barrier function was assessed, evaluating the intestinal permeability to 4 kDa-dextran. No changes were observed due to adipokine supplementations. This result in early life rats is in contrast to that found in two different studies; in one of them an increase in intestinal permeability after an intraperitoneal injection of leptin in adult rats was found (32), and, on the contrary, in an *in vitro* study a reduction in permeability was observed (33). The variability in these results highlights the importance of the type of approach (*in vitro* vs*. in vivo*) but also the state of development of the animals (suckling vs. adult).

To our knowledge, this is the first study to report the effect of adipokine supplementation on IEL composition. Although no changes were observed in Th or Tc TCRγδ^+^ cells, the three supplemented groups showed an increase of Tc TCRαβ^+^ and NKT cell percentages compared with the Reference group. These changes were also observed in our previous studies in other immune compartments, where the supplementation with both adipokines was able to enhance both subsets on the spleen (19) and T TCRαβ^+^ cells on the mesenteric lymph nodes (MLN) (20), suggesting an enhancement of immune maturation. However, a decrease in the NK cell proportion was detected after the three nutritional interventions in comparison to the Reference group. As has been described, there is a physiological depletion in NK IEL throughout the suckling period (13), and the enhancement in the reduction could indicate a promotion of NK maturation caused by the supplementations.

Regarding the proportion of cells bearing CD8, the three supplementations were able to increase their percentage. There is no information on the effect of these adipokines or similar components in the intestinal effector lymphocytes, but previously, in leptin-supplemented rat pups, an increase of CD8^+^ proportion in both MLN and spleen was reported (19, 20). Moreover, adiponectin and WPC administration also increased CD8^+^ cells in the MLN (20) and spleen (19), respectively. Siegmund et al. observed that *ob/ob* mice had fewer CD8^+^ in the IEL population and these cells also produced less IFN-γ compared to wild-type mice (34), contributing to an increase of the infections due to their role modulating the immunity toward microbes (35). This fact together with our present and previous results, strengthens the leptin role in promoting the main effector cells in contact with the lumen (CD8^+^ IEL). Interestingly, both adipokines act in different ways, thus adiponectin and WPC supplementation increased the percentage of CD8αβ^+^ cell subset, whereas leptin raised that of CD8αα^+^ cell subset, leading to an increase in the CD8αα^+^/CD8αβ^+^ ratio in the Leptin group compared to the Reference group. The increase in CD8αα^+^ cell proportion could be beneficial because this phenotype is more characteristic at intestinal level (35), suggesting a local effect resulting from leptin supplementation. Moreover, the three dietary interventions did not modify the integrin αE^+^, CD62L^+^, or CD25^+^ cell percentage after 21 days of supplementation, suggesting that they do not have an impact on the intestinal homing or activation pathways.

To further understand the impact of the studied supplementations on the intestinal immune system, the intestinal gene expression of some key molecules was assessed. No effect was observed in the gene expression of the analyzed TLR due to leptin or WPC supplementation, whereas a decrease in TLR-2 and TLR-5 gene expression was observed due to adiponectin supplementation. TLR-2 has an important role in the recognition of molecules present in the membrane of multiple pathogens, such as bacteria, virus, fungi and parasites (36), whereas, TLR-5 detects the flagella of bacteria (37). Thus, the decrease of these intestinal TLR could be related to the anti-inflammatory properties attributed to adiponectin (38–40).

The TNF-α cytokine and the mucines, MUC-2, and MUC-3, present in the small intestine act as an innate defense against different pathogens, and a lack of these components result in increased bacterial adhesion (41). MUC-2 gene was responsible for the gel-forming mucins, whereas MUC-3 gene makes membrane-bound mucins (42). Moreover, TNF-α regulates the mucus layer, increasing expressions of pIgR and MUC-2; it modulates tight-junctions, increasing intestinal permeability, induces cell death, restitutes and regenerates the epithelium, increasing cell migration and proliferation and interacts with the immune system (43). The supplementation with leptin or adiponectin during 21 days was able to increase the gene expression of these three molecules compared to the Reference group, suggesting a barrier reinforcement due to adipokines in early life. The effect on MUC-2 was the only one found already in the middle of the suckling period. In line with these results, it has been described in rats that a perfusion of leptin was able to increase the expression of MUC-2 and MUC-3 and to stimulate mucus-secreting goblet cells (44, 45). However, there is no information regarding whether adiponectin affects these intestinal genes or modulates the intestinal epithelial barrier. Gene expression of pIgR, Blimp-1 and FcRn is related to intestinal function and maturation because their expression changes during the suckling period. Whereas, the intestinal pIgR is barely expressed during the suckling and increases after weaning (46), the Blimp-1 and FcRn are highly expressed in early life and decrease at weaning (47). In our study, although the adipokines supplementation did not modify the proportion of pIgR, they were able to decrease the gene expression of FcRn on day 21, suggesting an early intestinal maturation. Unexpectedly, a punctual increase on Blimp-1 mRNA levels was observed after leptin supplementation. This fact triggers an intricateness interpretation because Blimp-1 expression has been described as being differential according to the intestinal section evaluated (47).

The study of the gene expression related to tight junction molecules showed that the adipokine supplementation did not produce any significant change in any of the studied days compared to the Reference group. Supporting this result, it has been reported that, in mice, after an intraperitoneal administration of 2 μg/g of adiponectin, no change in colonic Occludin and ZO-1 protein abundance at day 10 of life was observed (33). Nevertheless, Kim et al. detected a decrease in Occludin gene expression after the addition of 100 ng/mL of leptin to Caco-2 BBe cell culture; however, it has to be taken into account that these *in vitro* results should be confirmed *in vivo* (48).

The effect of adipokine supplementation during the whole suckling period on the gut microbiota composition was also studied. The three studied components were able to decrease the Proteobacteria phylum proportion. It has been described that breast-fed infants had a lower abundance of Proteobacteria than those fed with formula (49). In addition, in overweight/obese mothers, who have high leptin concentration in their breast milk, a lower abundance of Proteobacteria phylum in the infant intestinal microbiota at 2 weeks old has also been observed (50). Those results, together with ours, suggest that leptin and adiponectin, adipokines present in breast milk, might be components involved in the growth regulation of this phylum in breast-fed infants. However, Rajala et al. described that leptin receptor deficient mice (*db/db*) showed lower Bacteroidetes and higher Firmicutes proportions than wild-type mice, results not detected in the present study (51). Overall, in the early life context and taking into account that in rats aged from 7 days old to 28 days old, the percentage of Proteobacteria naturally decreases (27). So here, the reduction triggered by the adipokine supplementation in this phylum could be related to an enhancement of the gut microbiota maturation. Furthermore, Proteobacteria was also related to inflammation and it is a risk factor for necrotizing enterocolitis development in infants, thus a depletion of this phylum could have a protective role against this common pediatric disease (10).

Although previous studies are focused on modifications at the phylum level, to our knowledge this is the first study to report the effect of adipokines on a deeper level of taxonomy. The decrease in the Proteobacteria phylum proportion was due to different changes at family level. The three evaluated supplementations were able to decrease the unclassified family from the *RF32* order. In addition, the leptin and WPC supplementations produced a lower percentage of *Desulfovibrionaceae* and leptin nutritional intervention was the only one that decreased the *Alcaligenaceae* family. Moreover, the leptin administration during 21 days was able to increase the *Ruminococcaceae* proportion and also the unclassified family from the *Clostridiales* order. In line with these results, Zhou et al. found a positive correlation between plasmatic leptin and the *Clostridiales* order in male adult mice (52). On the other hand, WPC was able to increase the *Prevotellaceae* and *Lactobacillaceae* families, whereas a decrease in *Lachnospiraceae* was detected. In accordance with that, increased levels of *Lactobacillus* due to whey protein intake were observed, specifically in obese donors (53). However, another study reported in chicks, among other changes, an increase in the relative proportion of the *Lachnospiraceae* and *Bacteroidetes* families in the cecal microbiota after WPC administration (54).

Regarding the genus level, 16S rRNA gene sequencing revealed a decrease in the *Blautia* genus in all supplemented groups. Low levels of *Blautia* have been observed in children with type 1 diabetes (55) or with Crohn's disease (56, 57). Moreover, rats supplemented with leptin showed higher *Clostridium* and *Dehalobacterium* genera and lower levels of *Holdemania* and *Sutterella*. Although Queipo-Ortuño et al. described a negative correlation between serum leptin and *Clostridium* genus (58), a higher concentration of the *Sutterella* genus was observed in infants with autism (59, 60). Further studies considering endocrine-gut-brain and leptin could be conducted to better understand the role of this adipokine in this process. Moreover, the adiponectin supplementation increased the proportion of the *Enterococcus* genus and decreased the *Roseburia* and *Allobacterium* genera. It is known that *Enterococcus* is an opportunistic bacteria that has been associated with human celiac disease (61); however, this is a preclinical study and further studies are needed to shed light on this field. With regard to the WPC supplementation, it was able to reduce the percentage of the *Anaerotruncus* and *Adlercreutzia* genera and to increase the proportion of the *Dehalobacterium* genus. Little is known about the possible role of the *Anaerotruncus* genus, but Lau et al. described a bacteraemia caused by *Anaerotruncus colihominis* (62), so the fact that the WPC decreased this genus might suggest a protective role in the newborn. Furthermore, children with inflammatory bowel disease or patients with multiple sclerosis showed a lower presence of *Adlercreutzia* compared with their respective controls (63, 64).

On the other hand, other changes have been observed due to leptin supplementation, such as new bacteria colonized the cecal flora (*Candidatus arthromitus*) and after WPC administration the genus *Lactococcus* was detected.

The importance of the interaction between both the intestinal immune system and microbiota is well-described. So, some of the modifications in IEL composition or gene expression could be due to the microbiota or vice-versa. For example, in mice it has been reported that for the expansion of T cells from the TCRαβ lineage, a normal microbial colonization of the intestine is required (15). Moreover, *Clostridia* species showed an important role in inducing Treg in the colonic mucosa of mice (65). On the other hand, adaptive immunity seems to modulate microbiota composition, as observed in B-cell-deficient mice, where they had alterations in their microbiota, making it less diverse. In addition, secretory IgA could modulate commensal bacteria and also gut microbiota induce its secretion, this being an example of the crosstalk between both of them (66, 67). It has also been described that changes in mucin composition could alter microbiota pattern and conversely, bacteria and their metabolites could modify the mucus layer or even degrade it (68).

In addition to the composition of the microbiota, their metabolites could also affect the immune system, and some of the effects found in this work may be due to the changes in the production of microbial metabolites due to the dietary interventions with the adipokines. For example, short chain fatty acids (SCFA), the most abundant molecules produced by microbiota, have been described as being able to suppress inflammation and increase Treg cells (69). Moreover, it has been described that Lactobacilli tryptophan metabolites are ligands of the aryl hydrocarbon receptor (AhR), which is a receptor involved in the homeostasis of IEL (70). With reference to adipokines, little is known about their effect on the microbial metabolites. However, the mechanisms involved are even more difficult to interpret as it was reported that SCFA could stimulate leptin production and adiponectin expression *in vitro* (71, 72). Overall, after demonstrating the influence of both adipokines on microbiota composition, their metabolism should be explored in terms of their impact on IEL development.

In conclusion, this study demonstrates that both leptin and adiponectin supplementation throughout the suckling period are able to modify the IEL composition by means of increasing Tc TCRαβ^+^, NKT, and CD8^+^ lymphocyte proportions and decreasing that of NK cells. Moreover, the intestinal innate immunity was also enhanced by the increase in TNF-α, MUC-2, and MUC-3 gene expression due to leptin and adiponectin intervention. These adipokines also play a role in the maturation of the intestine by the early reduction of the FcRn expression. In addition, we have reported for the first time the effect of leptin and adiponectin on the gut microbiota composition at the end of the suckling period. Overall, this study reinforces the role of leptin and adiponectin in enhancing the development of the effector immune system and microbiota in the intestinal compartment.

## Data Availability Statement

The raw data supporting the conclusions of this manuscript will be made available by the authors, without undue reservation, to any qualified researcher.

## Ethics Statement

The studies were performed in accordance with the criteria outlined by the Guide for the Care and Use of Laboratory Animals. Experimental procedures were reviewed and approved by the Ethical Committee for Animal Experimentation of the University of Barcelona and the Ethical Committee of the Generalitat de Catalunya (CEEA/UB 220/15 and DAAM: 8521).

## Author Contributions

AF, MC, MR-L, and FP-C conceived and designed the experiments. BG-P, SB, OP, and FF analyzed microbiota composition. BG-P wrote the initial draft of the paper. All authors carried out the experiments and the data analysis, and were involved in the interpretation of the data and critical revision of the manuscript, and have read and approved the final version of the manuscript for publication.

### Conflict of Interest

The authors declare that the research was conducted in the absence of any commercial or financial relationships that could be construed as a potential conflict of interest.

## Supplementary Material

Supplementary Figure 1 Intestinal permeability to 4 kDa-FITC-dextran **(A)** and intestinal gene expression of MUC-2, MUC-3, Blimp-1, FcRn, Claudin-2, Claudin-4, ZO-1, and Occludin **(B)** at day 10 from the four groups: Reference, Leptin, Adiponectin, and Whey Protein Concentrate (WPC) (*n* = 8–12 pups). Statistical differences: ^*^*p* < 0.05 vs. Reference group.
