# Supplementary figures and images for: Influence of Leptin and Adiponectin Supplementation on Intraepithelial Lymphocyte and Microbiota Composition in Suckling Rats
Source: Front Immunol. 2019 Oct 9;10:2369. doi: 10.3389/fimmu.2019.02369 (PMC6795087; doi:10.3389/fimmu.2019.02369)

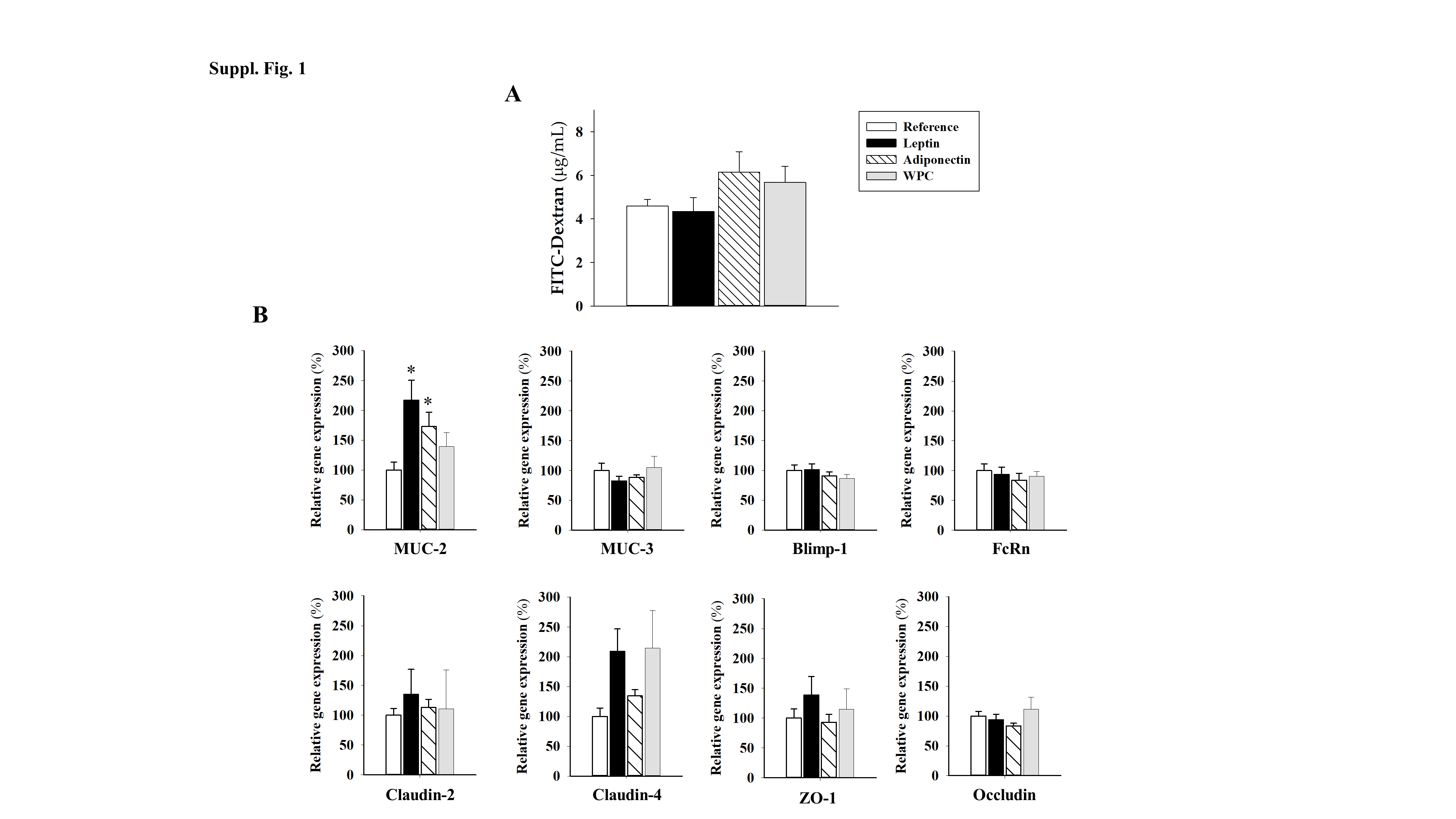

Supplement: Supplementary Figure 1 — Intestinal permeability to 4 kDa-FITC-dextran (A) and intestinal gene expression of MUC-2, MUC-3, Blimp-1, FcRn, Claudin-2, Claudin-4, ZO-1, and Occludin (B) at day 10 from the four groups: Reference, Leptin, Adiponectin, and Whey Protein Concentrate (WPC) (n = 8–12 pups). Statistical differences: *p < 0.05 vs. Reference group. [file Image_1.TIF]
